# Supplementary material for: Polyketide synthase gene clusters in the ericoid mycorrhizal fungus Oidiodendron maius: genome-wide prediction, expression profiling, and functional characterization of knockout mutants
Source: Front Plant Sci. 2026 Sep 16;17:1924430. doi: 10.3389/fpls.2026.1924430 (PMC13623903; doi:10.3389/fpls.2026.1924430)
Supplement: Supplementary file 1 [file SupplementaryFile1.zip › Supplementary Materials/Supplementary Folder 1.zip!/Oidma1_AssemblyScaffolds_Repeatmasked/knownclusterblast/cluster39/jgi.p_Oidma1_132717_mibig_hits.html]

| MIBiG Protein | Description | MIBiG Cluster | MiBiG Product | % ID | % Coverage | BLAST Score | E-value |
| --- | --- | --- | --- | --- | --- | --- | --- |
| CBF80711.1 | TdiA\_[Source:UniProtKB/TrEMBL;Acc:A7XRY0] | BGC0000442 | Terrequinone\_biosynthetic\_gene\_cluster | 40 | 104.180064309 | 671 | 1.8e-192 |
| AHN91924.1 | EchA | BGC0000340 | Echosides\_biosynthetic\_gene\_cluster | 35 | 101.714898178 | 581 | 2.4e-165 |
| ACH90386.1 | atromentin\_synthetase | BGC0001256 | Atromentin\_biosynthetic\_gene\_cluster | 35 | 97.7491961415 | 531 | 2.2e-150 |
| AAO62585.1 | peptide\_sythetase\_polyketide\_synthase\_fusion\_protein | BGC0001016 | Microcystin\_biosynthetic\_gene\_cluster | 29 | 68.4887459807 | 262 | 2.5e-69 |
| AAF00957.1 | McyG | BGC0001017 | Microcystin\_biosynthetic\_gene\_cluster | 28 | 71.5969989282 | 256 | 1.4e-67 |
| CAD29795.1 | peptide\_synthetase | BGC0001015 | Microcystin\_biosynthetic\_gene\_cluster | 27 | 71.7041800643 | 252 | 2.6e-66 |
| ABM21569.1 | CrpA | BGC0000975 | Cryptophycin\_biosynthetic\_gene\_cluster | 27 | 71.4898177921 | 242 | 1.6e-63 |
| CAG23957.2 | NRPS/PKS\_protein | BGC0001089 | Bacillaene\_biosynthetic\_gene\_cluster | 27 | 71.1682743837 | 242 | 2.1e-63 |
| ABM21572.1 | CrpD | BGC0000975 | Cryptophycin\_biosynthetic\_gene\_cluster | 29 | 60.5573419078 | 238 | 3e-62 |
| CAM59601.1 | non-ribosomal\_peptide\_synthetase/polyketide\_synthase\_hybrid\_enzyme | BGC0000297 | Aeruginoside\_biosynthetic\_gene\_cluster | 28 | 64.0943193998 | 210 | 8.7e-54 |
| ACM68682.1 | AerA | BGC0000298 | Aeruginosin\_biosynthetic\_gene\_cluster | 28 | 64.308681672 | 206 | 1.3e-52 |
| CAF05650.1 | TubE\_protein | BGC0001053 | Tubulysin\_biosynthetic\_gene\_cluster | 26 | 67.2025723473 | 206 | 1.6e-52 |
| ABS74181.1 | BmyA | BGC0001090 | Bacillomycin\_biosynthetic\_gene\_cluster | 23 | 69.667738478 | 161 | 3.5e-39 |
| BAB69698.1 | iturin\_A\_synthetase\_A | BGC0001098 | Iturin\_biosynthetic\_gene\_cluster | 23 | 69.667738478 | 161 | 6e-39 |
| ADE34493.1 | SsfL2 | BGC0000269 | SF2575\_biosynthetic\_gene\_cluster | 25 | 42.0150053591 | 90 | 1.3e-17 |
| ABS74201.1 | YngI | BGC0001095 | Fengycin\_biosynthetic\_gene\_cluster | 28 | 32.9046087889 | 84 | 9.4e-16 |
| AFU65902.1 | DacH | BGC0000216 | Dactylocycline\_biosynthetic\_gene\_cluster | 28 | 31.2968917471 | 82 | 2.1e-15 |
| AAC06348.1 | bacitracin\_synthetase\_3 | BGC0000310 | Bacitracin\_biosynthetic\_gene\_cluster | 24 | 47.9099678457 | 81 | 4.7e-15 |
| AHD25943.1 | ChdL | BGC0000208 | Chelocardin\_biosynthetic\_gene\_cluster | 25 | 31.2968917471 | 74 | 7.4e-13 |
| AAF86395.1 | FkbP | BGC0000994 | FK520\_biosynthetic\_gene\_cluster | 22 | 53.4833869239 | 73 | 1.7e-12 |
| ADY16697.1 | TqaA | BGC0001142 | Tryptoquialanine\_biosynthetic\_gene\_cluster | 23 | 54.7695605573 | 72 | 3.7e-12 |
| AAC68816.1 | FK506\_peptide\_synthetase | BGC0000353 | FK506\_biosynthetic\_gene\_cluster | 22 | 53.6977491961 | 69 | 2.4e-11 |
| BAG17643.1 | putative\_NRPS-type-I\_PKS\_fusion\_protein | BGC0001043 | SGR\_PTMs\_biosynthetic\_gene\_cluster | 27 | 22.293676313 | 67 | 1.2e-10 |
| ACG60773.1 | NRPS(C/A/PCP) | BGC0001058 | Zorbamycin\_biosynthetic\_gene\_cluster | 23 | 53.3762057878 | 67 | 1.2e-10 |
| CAA73127.1 | HMWP1\_protein | BGC0000467 | Yersiniabactin\_biosynthetic\_gene\_cluster | 23 | 28.2958199357 | 66 | 2.6e-10 |
| CAJ87591.1 | putative\_peptide/polyketide\_synthetase | BGC0001055 | Yersiniabactin\_biosynthetic\_gene\_cluster | 23 | 28.1886387996 | 65 | 3.5e-10 |
| ABR67758.1 | CmnO | BGC0000316 | Capreomycin\_biosynthetic\_gene\_cluster | 23 | 34.1907824223 | 63 | 1.7e-09 |
| BAB69700.1 | iturin\_A\_synthetase\_C | BGC0001098 | Iturin\_biosynthetic\_gene\_cluster | 25 | 26.3665594855 | 62 | 2.2e-09 |
| CAY48788.1 | putative\_non-ribosomal\_peptide\_synthetase | BGC0001312 | Viscosin\_biosynthetic\_gene\_cluster | 25 | 29.5819935691 | 57 | 9.4e-08 |
| BAC67535.1 | arthrofactin\_synthetase\_B | BGC0000305 | Arthrofactin\_biosynthetic\_gene\_cluster | 25 | 29.6891747053 | 56 | 2.1e-07 |
| AOA33121.1 | nonribosomal\_peptide\_synthetase | BGC0001346 | Bananamides\_biosynthetic\_gene\_cluster | 22 | 35.0482315113 | 54 | 8e-07 |
| CAI94706.1 | putative\_aminocoumarin\_ligase | BGC0000141 | Rubradirin\_biosynthetic\_gene\_cluster | 26 | 23.3654876742 | 54 | 1e-06 |
| ABL86391.1 | hybrid\_polyketide\_synthase\_and\_nonribosomal\_peptide\_synthetase | BGC0000999 | Heat-stable\_antifungal\_factor\_biosynthetic\_gene\_cluster | 23 | 31.6184351554 | 53 | 1.4e-06 |
